# Remote Sensing Image Classification Using the Spectral-Spatial Distance Based on Information Content

**DOI:** 10.3390/s18103428

**Published:** 2018-10-12

**Authors:** Siya Chen, Hongyan Zhang, Tieli Sun, Jianjun Zhao, Xiaoyi Guo

**Affiliations:** 1 School of Geographical Science& School of Information Science and Technology, Northeast Normal University, Changchun 130024, China; chensy413@nenu.edu.cn (S.C.); zhaojj662@nenu.edu.cn (J.Z.); guoxy914@nenu.edu.cn (X.G.); 2 Key Laboratory of Intelligent Information Processing of Jilin Universities, Northeast Normal University, Changchun 130024, China

**Keywords:** remote sensing image classification, statistical region merging, information content, contextual classifier

## Abstract

Among many types of efforts to improve the accuracy of remote sensing image classification, using spatial information is an effective strategy. The classification method integrates spatial information into spectral information, which is called the spectral-spatial classification approach, has better performance than traditional classification methods. Construct spectral-spatial distance used for classification is a common method to combine the spatial and spectral information. In order to improve the performance of spectral-spatial classification based on spectral-spatial distance, we introduce the information content (IC) in which two pixels are shared to measure spatial relation between them and propose a novel spectral-spatial distance measure method. The IC of two pixels shared was computed from the hierarchical tree constructed by the statistical region merging (SRM) segmentation. The distance we proposed was applied in two distance-based contextual classifiers, the k-nearest neighbors-statistical region merging (k-NN-SRM) and optimum-path forest-statistical region merging (OPF-SRM), to obtain two new contextual classifiers, the k-NN-SRM-IC and OPF-SRM-IC. The classifiers with the novel distance were implemented in four land cover images. The classification results of the classifier based on our spectral-spatial distance outperformed all the other competitive contextual classifiers, which demonstrated the validity of the proposed distance measure method.

## 1. Introduction

Classification is an important technology to extract useful information from remote sensing images, so improving the accuracy of classification is always a popular research topic. The newest improvement direction focuses on exploiting spectral-spatial features to classify remote sensing images [1], namely spectral-spatial classification. Spectral-spatial classification approaches can also be called contextual classification approaches. Spectral-spatial classifiers are more accurate and work better compared with the traditional classifiers because they take into account the contextual spatial characteristics to enhance the performance of the classification results [2]. The techniques commonly used in contextual classifiers to extract spatial features include the random field (RF) [3], mathematical morphology [4], segmentation and clustering [5], and deep learning [6].

The random field (RF) is a useful approach to capture the prior knowledge of an image. It models the classification task as a RF model to obtain the spatial correlation between pixels and realize the spectral-spatial classification. The Markov random field (MRF) was first introduced into spectral-spatial classification and significantly improved the traditional classifiers. Numerous works can be found that combine MRF with various traditional classifiers, including Bayesian Network [7], the SVM [8,9] and the optimum-path forest (OPF) [10,11,12]. Later, the conditional random field (CRF) model as an improved MRF model was successfully applied in spectral-spatial classification [13]. CRF models the posterior distribution as more flexible in classification. Different from the MRF considers spatial features at the label level, the CRF incorporates the spatial information on both the pixel data level and the class label level [14]. With the appearance of new, powerful optimization algorithms that can solve CRF inference problems, the application of CRF is more extensive [15]. 

Classifying pixels that belong to one region as homogeneous is another typical way to consider spatial information. The region can be obtained by implementing segmentation and clustering on an image. One strategy is to perform segmentation (e.g., watershed (WH), the squared-error clustering method (ISODATA) and expectation maximization (EM)) and pixel-wise classification independently, and the final classification result is determined by the majority voting (MV) rule within the segmented region [16,17]. Another way is to segment the image using fractional-order Darwinian particle swarm optimization (FODPSO) and mean shift segmentation (MSS) first, and then the segmented image is used as the input of the pixel wise classification [18]. The third type is to construct the spectral-spatial similarity or spectral-spatial distance measure, such as Huo et al., who computed both the spatial and spectral similarity by hierarchical segmentation (HSEG) to construct the spatial kernel for the SVM [19]. 

The original mathematical morphology-based contextual classifier exploits morphological profiles (MPs) to represent the spectral-spatial information. The MP is built from the first principal components of the image and constructs an extended morphological profile (EMP) used for classification [20]. However, there are still some limitations in MPs that were overcome by attribute profiles (APs) [21]. APs are more flexible and efficient than MPs but suffer from the threshold values. The extended attribute profile (EAP) should be built for classification [22]. The extended multi attribute profile (EMAP) is obtained when several constructed EAPs considering different attributes are stacked together [23]. Recently, an advanced approach was proposed, named the extinction profile (EP), which is fully automatic and independent of the dataset’s distribution [24]. 

The capability of deep learning to extract joint spatial-spectral features independently has been proven in recent years and become a popular way to improve classification results [25]. Lee et al. [26] and Yang et al. [27] proposed deep convolutional neural network (CNN)-based contextual remote sensing image classification. By considering the size of the spatial window in the CNN as limited to 3*3, Chen et al. proposed a novel method that uses a larger spatial window to integrate features in the CNN [28]. Erchan et al. explored the collaboration of two powerful methods, APs and the CNN, to achieve a more effective strategy for classification [29].

In addition to the above methods, jointly taking into account the spatial and spectral information on dimensionality reduction of hyperspectral images (HSI) is also an effective way to achieve spectral-spatial classification. Yuan et al. [30] propose a spectral-spatial linear discriminant analysis (SSLDA) and its kernel version spectral-spatial kernel discriminant analysis (SSKDA). Sellami and Farah [31] incorporated the tensor locality preserving projections method (TLPP) and constrained band selection method (CBS) to preserve as much as possible the spectro-spatial information during spectro-spatial dimensionality reduction in HSI.

In this paper, we extend two segmentation-based spectral-spatial classifiers in [32] by propose a novel spectral-spatial distance measure method. Chen et al. proposed a spectral-spatial distance using statistical region merging (SRM) segmentation for two distance-based classifiers, the k-NN and OPF, and obtained two contextual classifiers, the OPF-SRM and k-NN-SRM. However, they only simply used the height information to compute the spectral-spatial distance ignored other useful information within segmentation results. In our novel spectral-spatial distance measure method, additional IC is also considered. The novel spectral-spatial distance measure method takes full advantage of spatial information in the segmentation results which makes the distance computation more accurate. Exploiting the proposed spectral-spatial distance in classification could reduce the effect of segmentation results on classification to some extent; make up for the shortcoming that the results of spectral-spatial classification method based on segmentation is highly dependent on segmentation. As far as we know, this is the first time to introduction IC in remote sensing image classification. Our improved spectral-spatial distance measure is applied to the OPF-SRM and k-NN-SRM to obtain two extended contextual classifiers, OPF-SRM-IC and k-NN-SRM-IC. The classification results using the improved measure on four real land cover remote sensing images outperformed the original classifiers and other competitive contextual classifiers, which illustrated the validity of the proposed spectral-spatial distance function. This paper is organized in five sections. Section 2 described the proposed spectral-spatial distance measure that considers the detailed IC. Section 3 presented the datasets used for the experiments and the parameters used in the experiments. Section 4 provided a discussion and Section 5 is the conclusion of this paper.

## 2. Materials and Methods

### 2.1. Principle of SRM Segmentation 

The SRM [33] is a commonly used hierarchical multiscale segmentation method. SRM is also a region-based segmentation method. The main idea of the SRM is to start with a pixel of an image and then determine whether a neighborhood should be added according to a particular order and merging criteria. The regional growth is stopped and image segmentation is completed when a certain condition is satisfied. The key steps in the SRM include the calculation of the merging prediction and merging sequence. 

Given $I^{*}$ is the real scene, the statistical regions within $I^{*}$ represent the theoretical true objects. Image I denotes the observation of $I^{*}$. Defined |⋅| is the cardinality of image, so there are |I| pixels contained in I in total. Each pixel of an image contains red-green-blue (RGB) values, each of the three belonging to the set {1,2,…,g}. In 8 bit images, g =256. Then each color channel of the pixels in $I^{*}$ could be described by a set of Q independent random variables within the range of [0, g/Q], which ensure the sum of the Q independent random variables belongs to {1,2,…,g}. The merging criterion is that the pixels inside a region are homogeneous if they have the same expected value for any given color channel, and the pixels that belong to different regions are separate if the expected values are different for at least one color channel. The merging predicate can be written as follows:

(1) $$P\left(R,\;R\prime \right)=\{\begin{array}{c} \mathrm{true}\;\mathrm{if}\;\forall \alpha \in \left\{R,G,B\right\},\left|\bar{R_{\alpha }^{’}}-\bar{R_{\alpha }}\right|\leq \sqrt{b^{2}\left(R\right)+b^{2}\left(R\prime \right)}\\ \mathrm{false}\;\mathrm{otherwise} \end{array},$$

where R and R′ are two adjacent candidate regions to be merged. $\bar{R_{\alpha }}$ is the mean value of region R in channel α. b(R) is calculated as follows:

(2) $$b\left(R\right)=g\sqrt{\frac{1}{2Q\left|R\right|}\left(\ln\frac{\left|S_{\left|R\right|}\right|}{\delta }\right)},$$

where $S_{\left|R\right|}$ denotes the set of regions with |R| pixels and $\delta =1/\left(6{\left|I\right|}^{2}\right)$. The regions R and R′ that are homogeneous will be merged if the P(R, R′) is true.

A pixel P and its 4 connecting neighbors (up, down, left and right), P′, constitute a pixel pair (P, P′). There are a total of N<2|I| pixel pairs. The SRM defines the merging sequence as the ascending order of dissimilarity of these pixel pairs. The pixel pair with the smallest dissimilarity is the first to be predicated. The dissimilarity is measured by $\left(P,\;P\prime \right)=\max\left|P_{\alpha }^{’}-P_{\alpha }\right|$, in which $P_{\alpha }$ and $P_{\alpha }^{’}$ are the values of pixels P and P′ in channel α∈{R,G,B}, respectively. So we can see, Q is the only parameter in SRM used to determine the statistical complexity of $I^{*}$. One can achieve different segmentation granularity by adjusting Q values. 

### 2.2. Spectral-Spatial Distance Based on SRM

As mentioned above, the merging prediction of the SRM segmentation method is based on the adjacent regions, and thus, the segmentation results of the SRM method reflect the spatial relation among the samples of an image. Moreover, the segmentation results of SRM could be used to measure the spatial distance between samples of an image.

**Figure 1:**
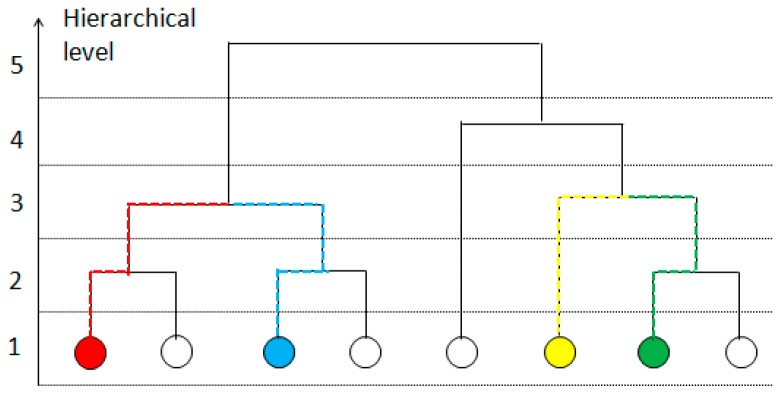
Example of a hierarchical tree.

In the segmentation results of SRM, the segmented regions decrease, and the number of regions becomes larger when Q increases and vice versa. We can obtain different scales of segmentation results of an image by tuning the Q values and put all these results together can build a hierarchical tree with the hierarchical level (HL) h. Such as in Figure 1, the hierarchical tree is constructed with the segmentation results of four different Q values for one image. Each level in the hierarchical tree is a different scale of segmentation. With the increasing values of Q, the hierarchical value levels decrease, the number of regions increases, and the size of regions decreases. The highest hierarchical level is H, in which is the initial undivided image. The structure of the hierarchical tree can be used to measure the spatial relation between the pixels. Huo et al. exploited the levels, namely the heights of segments to compute the spatial similarity between pixels. The segment with the minimum level among all common segments of two pixels calls their first common segment. The hierarchical level of the first common segment of pixels $x_{i}$ and $x_{j}$ is computed as their hierarchical level distance $d_{\mathrm{HL}}$. Then the similarity formula is defined as follows:

(3) $${\mathrm{Sim}}_{\left(x_{i},x_{j}\right)}=0.9^{ad_{\mathrm{HL}}\left(x_{i},x_{j}\right)},\;\left(a>0\right),$$

(4) $$d_{\mathrm{HL}}\left(x_{i},x_{j}\right)=\underset{1\leq h\leq H}{\mathrm{argmin}}\left\{{\mathrm{seg}}_{i}^{h}={\mathrm{seg}}_{j}^{h}\right\},$$

where ${\mathrm{seg}}_{i}^{h}$ denotes that pixel $x_{i}$ belongs to a segment at level h. According to the above formula, the hierarchical level distance $d_{\mathrm{HL}}$ of the green and yellow objects in Figure 1 is 3. The lower the hierarchical level h of the first common segment of two pixels is, the more spatially similar they are. The spatial similarity is minimal when the first common segment of two pixels is the original undivided image at the highest level H. 

The traditional distance function is computed based on the spectral feature space when applied in remote sensing image classification, which ignore the spatial characteristics useful for classification and lead to a lower classification accuracy. Inspired by Huo, Chen et al. proposed a spectral-spatial distance based on SRM, which considered both the spatial and the spectral characteristics of remote sensing image during classification. They used $1/0.9^{d_{\mathrm{HL}}\left(x_{i},x_{j}\right)}$ to denote the spatial distance $f_{\mathrm{spatial}}$ between pixels $x_{i}$ and $x_{j}$ and combined it with the traditional spectral feature to get a hybrid spectral-spatial distance function as follows:

(5) $$d_{\mathrm{spectral}-\mathrm{spatial}}\left(x_{i},x_{j}\right)=f_{\mathrm{spatial}}d_{\mathrm{spectral}}\left(x_{i},x_{j}\right),$$

where $d_{\mathrm{spectral}}$ is the distance between pixels based on the spectral feature space, such as the Euclidean distance that was used. 

### 2.3. Novel Spectral-Spatial Distance Based SRM Considering IC

The accuracy of classification based on spectral-spatial distance proposed by Chen et al. is highly dependent on the performance of the SRM segmentation results, so we should make full use of the available information within the segmentation results and the corresponding hierarchical tree. However, Chen et al. only simply used the height information of segments in the hierarchical tree, and there is still a lot of other information of hierarchical tree can used to capture richer spatial characteristics and more accurate spatial relation. In this section, we extend the works of Chen et al. by proposing an improved spectral-spatial distance measure. The proposed distance measure method introduced the IC of two pixels shared.

The notion of IC was widely applied in the concept similarity measure method in data mining. Following the standard argumentation of information theory, the IC of a concept can be quantified by the probability. As the probability increases, the more abstract a concept becomes and the lower its IC. This same argument can apply to segments in a segmented image. The more pixels a segment contains increases its probability and lowers its IC. In the hierarchical tree obtained by SRM, from the low hierarchical level to the high hierarchical level, the size of segments goes from small to large, the segments go from specific to abstract, and the IC in segments goes from large to small. According to Resnik [34], the similarity between concepts depends on the information that two concepts shared, which could be computed by the IC of their common ancestor. Inspired by Resnik, we can obtain a new way to measure pixel distances through the quantitative characterization of information. The distance between the two pixels depends on the information that the two pixels shared. The less information that two pixels share, the more dissimilar they are, and the greater the distance. 

The distance measure-based IC between pixels is defined as follows:

(6) $$d_{\mathrm{IC}}=log\left(count_{\mathrm{FCS}}\left(x_{i},x_{j}\right)\right)/log\left(N\right),$$

where $count_{\mathrm{FCS}}\left(x_{i},x_{j}\right)$ is number of pixels in the first common segment of $x_{i}$ and $x_{j}$. N is the total number of pixels the image contains. This fraction normalizes the $d_{\mathrm{IC}}$ value between 0 and 1. According to function (6), the distance of a pixel from itself is 0. The distance is at the maximum of 1 when the first common segment of two pixels is the initial undivided image at the maximum hierarchical level H. When the first common segment of the two pixel points contains more pixels, these two pixels share less information, and the distance between them is greater. Conversely, if the first common segment of two pixel points contains fewer pixels, the two pixels share more information, and the distance value is smaller. For example, the first common segment of the red and blue pixels and the first common segment of the yellow and green pixels are both at hierarchical level 3. However, the region sizes of these two first common segments are different. The first common segment of the red and blue pixels contains four pixels, and the first common segment of the yellow and green pixels contains three pixels. Therefore, the spatial similarity between them is different. We combine our distance-based IC with the $d_{\mathrm{spectral}-\mathrm{spatial}}$ proposed by Chen et al. to obtain a comprehensive distance formula:

(7) $$D=d_{\mathrm{spectral}-\mathrm{spatial}}\times d_{\mathrm{IC}},$$

**Figure 2:**
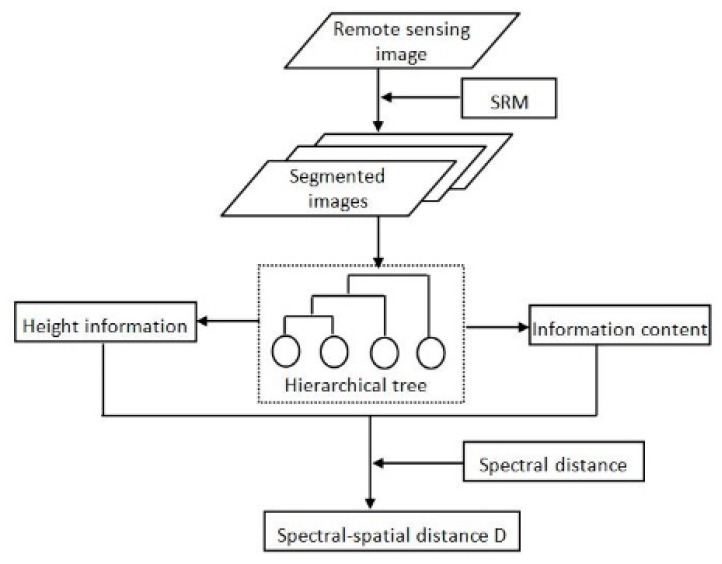
Flowchart of the proposed comprehensive spectral-spatial distance D.

Figure 2 is the flowchart of the proposed spectral-spatial distance-based SRM considering IC. The proposed comprehensive distance formula measures distance between sample both in spatial level and spectral level. Both the height information and IC are taken into account during spatial distance measurement which could increase the accuracy of the spatial distance and the final distance.

### 2.4. Application of the Proposed Comprehensive Distance Measure

The advantage of the distance-based remote sensing image classification algorithm is that it is simple and easy to execute, but the accuracy of the algorithm needs to be improved. Especially, the traditional distance function in this type classifier only use the spectral characteristic and only depend on the spectral distance between samples during the classification, which ignore the spatial characteristic of remote sensing data. This can be avoiding by applying the hybrid spatial-spectral distance. 

The k-NN and OPF algorithms are two commonly used methods based on distance due to their good performance. And they are closely related to each other. The OPF is equivalent to the 1-NN when all training samples are used as prototypes in the OPF [35]. Chen et al. applied their spectral-spatial distance to these two classifiers obtained two contextual classifiers, the OPF-SRM and k-NN-SRM. The comprehensive distance considers the IC proposed in this paper can also be applied to these two distance-based classifiers. We applied our comprehensive distance measures to the k-NN and OPF to obtained two contextual classifiers named as the k-NN-SRM-IC and OPF-SRM-IC, respectively. The comprehensive distance formula proposed in this paper is used to measure distance between samples in k-NN-SRM-IC and OPF-SRM-IC.

#### 2.4.1. k-NN-SRM-IC 

The idea of k-NN is each test sample can be represented and classified according to its *k*-nearest neighbors in the training set. The classifier assigns a test sample to the class that its *k*-nearest neighbors most belong to. The novel comprehensive distance formula considers IC is used to find the k-nearest neighbors in k-NN-SRM-IC.

#### 2.4.2. OPF-SRM-IC 

OPF construct a complete graph A that each pair of nodes is connected by a single edge in the training stage, the nodes of A is the samples of training set $Z_{1}$, and the weight of edge is the distance between the nodes connected by this edge. The adjacent nodes with different labels in the minimum spanning tree (MST) of graph A were chosen as prototypes set S; each prototype represents one class. $\pi_{t}$ denotes a path consist of a sequence of adjacent samples start from a root R(t) and end with sample t. The maximum distance between adjacent samples along $\pi_{t}$ defined as the path-cost $f\left(\pi_{t}\right)$, which reflects connection strength of nodes in path. The connection strength between R(t) and t is maximum and path $\pi_{t}$ is optimum when$\;f\left(\pi_{t}\right)\leq f\left(\tau_{t}\right)$, where $\tau_{t}$ is any other path containing node t. All the other samples in the training set are connected to a prototype with the minimum path-cost$\;f\left(\pi_{t}\right)$. And the minimization of $f\left(\pi_{t}\right)\;$is assigned to sample t as the minimum cost $C_{t}\;$used for the testing stage. A prototype with all the samples connected constitute an optimum tree, all the optimum trees constitute the optimum forest which is used to classified the testing set. 

For a given testing sample v in the testing set $Z_{2}$, OPF connect it to all the samples $t\in Z_{1}$. Then we find the optimum path P(v) according to the minimum cost $C_{v}$:

(8) $$\;C_{v}=\min\left\{\max\left\{C_{t},d\left(v,t\right)\right\}\right\},\;\forall t\in Z_{1},\;$$

The prototype included in P(v) is connected most closely with v and they are most likely within the same class, so label v with the class of R(v). The novel comprehensive distance formula considers IC is used to compute the weight of edge and the cost function in OPF-SRM-IC.

## 3. Results

### 3.1. Datasets

**Figure 3:**
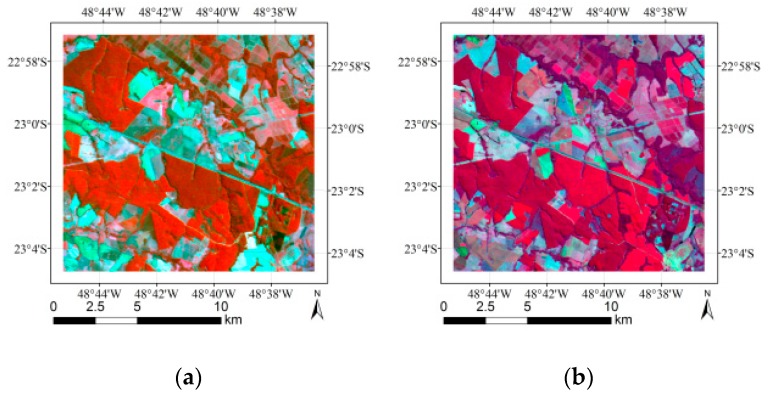

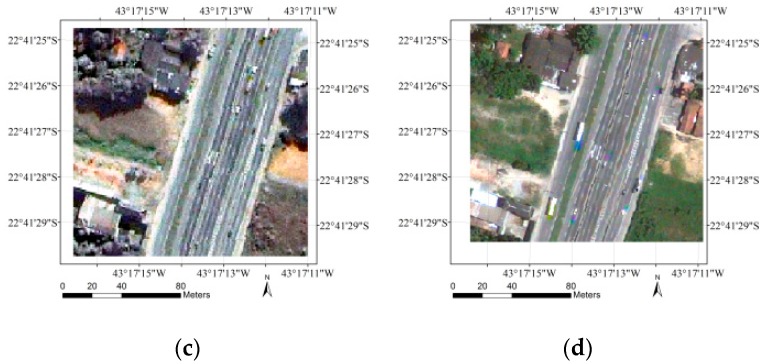
Images obtained from the (**a**) CBERS-2B CCD (20 m) sensor, band 2 (520–590 nm) = red, band 3 (630–690 nm) = green, band 4 (770–890 nm) = blue, (**b**) Landsat-5 TM (30 m) sensor, band 4 (760–900 nm) = red, band 3 (630–690 nm) = green, band 5 (1550–1750 nm) = blue, (**c**) Ikonos-2 MS sensor, band 4 (760–900 nm) = red, band 3 (630–690 nm) = green, band 2 (520–600 nm) = blue, and (**d**) Geoeye, band 5 (780–920 nm) = red, band 4 (655–690 nm) = green, band 3 (510–580 nm) = blue. The Ikonos-2 MS and Geoeye images were obtained through a fusion process between MS (4 m) and PAN (1 m), resulting in a final spatial resolution of 1 m.

**Figure 4:**
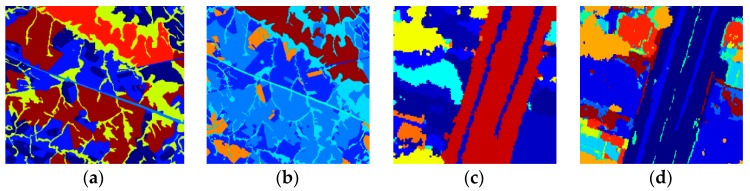
Ground truth images for (**a**) CBERS-2B, (**b**) Landsat-5, (**c**) Ikonos-2 MS and (**d**) Geoeye.

The datasets used in this paper are land cover images obtained from the CBERS-2B and Landsat-5 TM covering the Itatinga area of Brazil and land cover images obtained from Ikonos-2 MS and Geoeye covering the Duque de Caxias area of Brazil. The Green, Red and Near IR bands for CBERS-2B, Ikonos-2 MS and Geoeye were used in the experiment. Near IR, Red and SW IR bands for Landsat-5 TM were used in the experiment. These four images are shown in Figure 3. The RGB values were used as features to describe each pixel. The ground truth images of the datasets were annotated by Rodrigo José Pisani [12] and shown in Figure 4. Table 1 and Table 2 describe the detailed information of land cover classes included in the datasets.

### 3.2. Parameter Settings

In addition to comparisons with the original k-NN-SRM and OPF-SRM, the contextual classifiers using the proposed comprehensive distance (k-NN-SRM-IC and OPF-SRM-IC) were also compared against with k-NN, OPF, SVM, the spectral-spatial classifiers based on WH segmentation as well as the spectral-spatial classifiers based on MRF. For the MRF- based classifiers, we used the OPF-MRF proposed by Nakamura [10] and the SVM-MRF proposed by Osaku [12]. The original spectral-spatial classifier based on WH and MV is the SVM-WH-MV [17], which uses the SVM as the pixel-wise classifier performed on the original hyperspectral image first. Then, for every watershed region, all the pixels are assigned to the most frequent class within this region by MV approach. Other classifiers can also be used as the pixel-wise classifier. Thus, we built another two WH-MV-based spectral-spatial classifiers, the k-NN-WH-MV and the OPF-WH-MV, with the k-NN and OPF pixel-wise classifiers, respectively. 

We used the accuracy measure proposed by Papa to assess the classification results [36]. For the OPF, we used LibOPF [37]. For the MRF-based classifiers, we used the code obtained from [12], and for the k-NN and WH-MV-based methods we used our own implementation. The training and testing sets were constructed using the holdout method with 5% and 95%, respectively. Considering that the randomness of the holdout method may affect the results, the classification for each image was executed five times with different partitions. The final accuracy is the mean value of five iterations’ results. The number of iterations for the MRF-based methods is ten. For the SVM-based methods, the kernel is the radial basis function (RBF), and the relevant parameters have been optimized. k was chosen from the interval [1,10] according to the result having the maximum accuracy. We set fourteen different Q values for each image using the segmentation. They are presented in Table 3 with the corresponding hierarchical level. When Q increases to a certain extent, the result of the segmentation tends to stabilize, and we can determine the maximum value of Q.

### 3.3. Quantitative Results

The accuracy of the classification methods using the proposed distance measure and comparison methods for all images is shown in Table 4. The values in brackets are the parameter values for the relevant classifiers, including β used in two MRF-based classifiers and k with the best results in the k-NN-based classifiers. The spectral-spatial classifiers using the proposed comprehensive distance that considers the IC have an obvious improvement. The accuracy of SRM-based methods that consider IC is higher than SRM-based methods without IC. Among all the methods, the k-NN-SRM-IC has the best performance and obtained the highest accuracy. For each pixel-wise classifier, the SRM-based methods performed better than WH-MV-based methods, which indicate that SRM is more effective than WH in capturing spatial information. The k-NN-SRM outperformed the SVM-MRF in the case that the k-NN has similar performance to the SVM, and the OPF-SRM outperformed OPF-MRF. This illustrates that the spatial information obtained by the SRM is much more accurate than the spatial information obtained by the MRF. 

### 3.4. Visual Results

**Figure 5:**
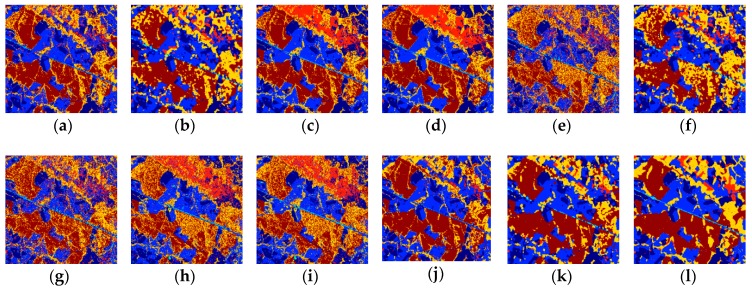
Classification results on the CBERS-2B image obtained by using the (**a**) k-NN, (**b**) k-NN-WH-MV, (**c**) k-NN-SRM, (**d**) k-NN-SRM-IC, (**e**) OPF, (**f**) OPF-WH-MV, (**g**) OPF-MRF, (**h**) OPF-SRM, (**i**) OPF-SRM-IC, (**j**) SVM, (**k**) SVM-WH-MV, (**l**) SVM-MRF.

Figure 5 shows the classification results for the CBERS-2B image. The k-NN-SRM-IC and k-NN-SRM obtained similar results that classified all the classes correctly, and the k-NN-SRM-IC is more accurate than the k-NN-SRM in culture. For the OPF-SRM and OPF-SRM-IC classifiers, reforesting and bushes were confused. The OPF failed to identify culture, reforesting and bushes. All the other methods cannot recognize culture completely. There are many discontinuous fragments in the results of methods using the WH and MV mechanisms.

**Figure 6:**
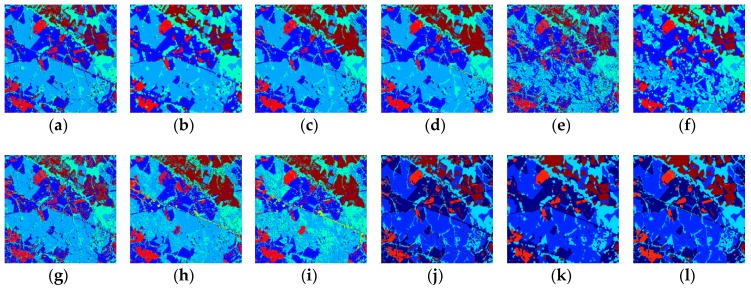
Classification results on the Landsat-5 image obtained by using the (**a**) k-NN, (**b**) k-NN-WH-MV, (**c**) k-NN-SRM, (**d**) k-NN-SRM-IC, (**e**) OPF, (**f**) OPF-WH-MV, (**g**) OPF-MRF, (**h**) OPF-SRM, (**i**) OPF-SRM-IC, (**j**) SVM, (**k**) SVM-WH-MV, (**l**) SVM-MRF.

As shown in Figure 6, the k-NN-SRM-IC and k-NN-SRM recognized all the classes correctly for the Landsat-5 image, and the k-NN-SRM-IC is more accurate than the k-NN-SRM in culture. The OPF, OPF-SRM, OPF-SRM-IC, and OPF-WH-MV confused a part of grasslands and reforesting. Other methods’ results are similar in that they misclassified a portion of reforesting and culture.

**Figure 7:**
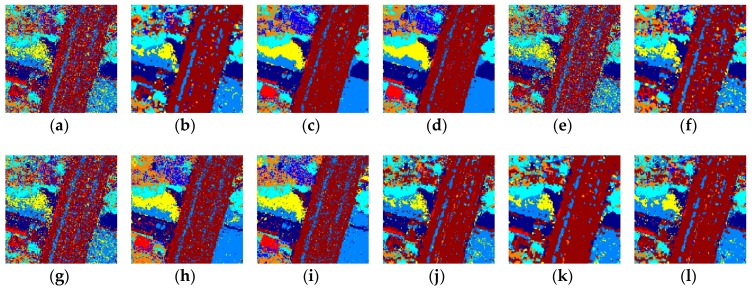
Classification results on the Ikonos-2 image obtained by using the (**a**) k-NN, (**b**) k-NN-WH-MV, (**c**) k-NN-SRM, (**d**) k-NN-SRM-IC, (**e**) OPF, (**f**) OPF-WH-MV, (**g**) OPF-MRF, (**h**) OPF-SRM, (**i**) OPF-SRM-IC, (**j**) SVM, (**k**) SVM-WH-MV, (**l**) SVM-MRF.

As shown in Figure 7, the result of the k-NN-SRM-IC is still the best for the Ikonos-2 MS image, and the k-NN-SRM is second. The OPF-SRM and OPF-SRM-IC misclassified bare soil as grassland. All the other methods misidentified tree coverage, clear tonal signatures and the covering of dark tonal signatures as road.

**Figure 8:**
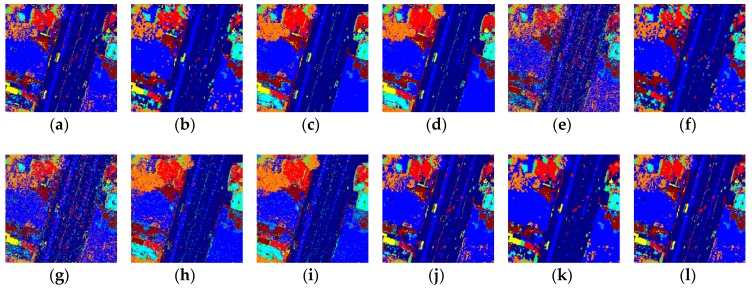
Classification results on the Geoeye image obtained by using the (**a**) k-NN, (**b**) k-NN-WH-MV, (**c**) k-NN-SRM, (**e**) k-NN-SRM-IC, (**e**) OPF, (**f**) OPF-WH-MV, (**g**) OPF-MRF, (**h**) OPF-SRM, (**i**) OPF-SRM-IC, (**j**) SVM, (**k**) SVM-WH-MV, (**l**) SVM-MRF.

As shown in Figure 8, the k-NN-SRM-IC classified all the classes correctly and achieved the best result on the Geoeye image once again. The k-NN-SRM can also distinguish all the classes, but the performance is poorer than the k-NN-SRM-IC in clear tonal signatures and bare, clear soil. The OPF-SRM and OPF-SRM-IC misclassified the clear tonal signatures. The k-NN, k-NN-WH-MV and OPF-MRF misclassified tree coverage and average tonal signatures. The OPF and OPF-WH-MV misclassified the clear tonal signatures, tree coverage and average tonal signatures. The SVM, SVM-MRF and SVM-WH-MV misclassified dark tonal signatures, tree coverage and average tonal signatures.

## 4. Discussion

The drawback of segmentation-based spectral-spatial classification methods is that they are highly dependent on the performance of the segmentation methods used, which includes two aspects: one is the accuracy of the segmentation results; the other is the information expression/capture capability of the segmentation results. The strategy based on MV belongs to the first case, the segmentation results are used directly to determine the final classification of samples. Once there are mistakes in the segmentation results, the final classification results will be affected. Such as the methods of combining WH and MV can improve the original classification to a certain extent, however, there are still many discontinuous fragments without practical significance. This is due to the WH segmentation results are over segmented.

In the methods of construct the spectral-spatial distance depending on segmentation results, the quality of spatial information captured from segmentation results affect the final classification results. Simply and insufficient spatial information express/capture from segmentation results may reduce the classification accuracy. In our proposed method, the spatial information capture capability of the segmentation results is enhanced. Our proposed method consider both the height information and the IC of samples to measure the spatial distance, which take full advantage of segmentation results and capture more complex spatial information. The performance of the k-NN-SRM-IC is better than the k-NN-SRM, and the OPF-SRM-IC outperformed the OPF-SRM in both quantitative and visual assessments, which illustrate the proposed comprehensive spectral-spatial distance considers IC is more effective than the spectral-spatial distance that only used the hierarchical level to capture spatial information. The results also indicate the feasibility and effectiveness that using the notion of the IC that two pixels share to measure the spatial distance between them is correct.

The classification results based on the MRF are complementary with the classification results based on the SRM. The classes that are correctly recognized by the MRF-based methods cannot be identified by SRM-based methods. Conversely, those classes that are identified correctly by the SRM-based methods are misclassified by the MRF-based methods. Thus, we can consider combining the MRF and SRM together to improve the classification in the future.

## 5. Conclusions

In this paper, we introduce IC to measure the spectral-spatial distance. We use the number of pixels contained in a segment to measure its IC. The more pixels that a segment includes, the more abstract that it is, and the less information the segment has. The fewer pixels that a segment includes, the more specific that it is, and the more information the segment has. The spatial distance between pixels can be computed according to the IC of their first common segment in a hierarchical tree constructed by the results of SRM multiscale segmentation. Integrating the spatial distance computed by the IC with the spectral-spatial distance that utilizes the hierarchical level and spectral features, we can obtain a novel spectral-spatial distance measure that can capture more spatial information. The novel spectral-spatial distance can be applied to the traditional distance-based classification method to improve the performance, such as the k-NN and OPF. The novel distance-based contextual classifiers using our novel spectral-spatial distance function, named the k-NN-SRM-IC and OPF-SRM-IC, are implemented in four land cover images. The k-NN-SRM-IC obtains the highest accuracy among all methods. The classification results proved the accuracy of the IC-based distance measure function and proved that the novel spectral-spatial distance function has the capability to capture more spatially related information between pixels. In the future, we will explore applying the proposed spectral-spatial distance in other contextual classifiers to improve the classification performance.

## Figures and Tables

**Table 1:** Colors corresponding to the six classes contained in the CBERS-2B and Landsat-5 images.

**Class**	grass lands	reforesting	cultures	roads	dams	bushes
**Color**	light green	dark gray	salmon	gray	red	green

**Table 2:** Colors corresponding to the eight classes contained in the Ikonos-2 MS and Geoeye images. A ninth class that exists in Geoeye is the average tonal signatures with the red color.

**Class**	roads	clear tonal signatures	bare moist soil	tree cover
**Color**	gray	white	salmon	dark green
**Class**	shadows	dark tonal signatures	bare soil clear	grasslands
**Color**	black	dark tonal signatures	yellow	light green

**Table 3:** The Q values and the corresponding hierarchical level values of h used in the SRM, and H=15.

Q	5	10	15	20	25	30	35	40	45	50	55	60	65	70
h	14	13	12	11	10	9	8	7	6	5	4	3	2	1

**Table 4:** The mean accuracy obtained by all methods on four land cover images.

Method/Image	CBERS-2B	Landsat-5	Ikonos-2MS	Geoeye
k-NN	69.95%(10)	71.43%(7)	68.84%(1)	75.32%(9)
k-NN-WH-MV	72.17%(1)	72.72%(1)	73.13%(1)	77.30%(1)
k-NN-SRM	76.90%(7)	76.69%(3)	79.49%(10)	82.45%(9)
**k-NN-SRM-IC**	**77.91%(7)**	**77.75%(3)**	**80.45%(10)**	**83.62%(9)**
OPF	66.04%	68.04%	68.00%	69.31%
OPF-WH-MV	70.74%	71.19%	72.27%	73.72%
OPF-MRF	68.90%(0.6)	71.11%(0.8)	69.42%(1.2)	72.97%(1.0)
OPF-SRM	73.65%	75.36%	78.84%	78.13%
OPF-SRM-IC	73.86%	76.83%	80.06%	79.02%
SVM	70.26%	71.21%	66.73%	76%
SVM-WH-MV	70.35%	71.40%	67.81%	76.47%
SVM-MRF	73.73%(1.2)	74.96%(1.3)	72.16%(1.2)	76.81%(1.3)
